# A Novel Fractal Coding Method Based on M-J Sets

**DOI:** 10.1371/journal.pone.0101697

**Published:** 2014-07-10

**Authors:** Yuanyuan Sun, Rudan Xu, Lina Chen, Ruiqing Kong, Xiaopeng Hu

**Affiliations:** 1 College of Computer Science and Technology, DaLian University of Technology, Dalian, China; 2 National Astronomical Observatories, Chinese Academy of Sciences, Beijing, China; Medical University of Graz, Austria

## Abstract

In this paper, we present a novel fractal coding method with the block classification scheme based on a shared domain block pool. In our method, the domain block pool is called dictionary and is constructed from fractal Julia sets. The image is encoded by searching the best matching domain block with the same BTC (Block Truncation Coding) value in the dictionary. The experimental results show that the scheme is competent both in encoding speed and in reconstruction quality. Particularly for large images, the proposed method can avoid excessive growth of the computational complexity compared with the traditional fractal coding algorithm.

## Introduction

Image compression provides an efficient way of digital image storage and transmission to reduce the irrelevance and redundancy of the image data. Among the various image compression techniques, fractal coding is an attractive one with its high compression ratio. Fractal image coding is based on the construction of an image transformation of a special kind which, when iterated on any initial image, produces a sequence of images that converges to a fractal approximation of the original [1]. Since this method was first proposed by Barnsley in 1988 [2], the method has been widely used in practical applications and has been improved upon by Jacquin [3]. In general, we call Barnsley’s method [4] as traditional fractal coding algorithm. Fractal image coding was used not only in image coding, but also in some interesting image problems, such as image retrieval [5]. However, compared with traditional image coding technologies, fractal compression suffers from the high computational complexity in encoding [6].

The most important thing in fractal coding research is to reduce the coding time without loss of restored image quality. In recent years, much work has been done on fractal compression. Qu et al. presented an algorithm combined with wavelet algorithm [7]. Duh et al. applied the DCT in fractal image compression [8]. Jaferzadeh et al. accelerated the compression speed by block classification [9]. Sze et al. used the quad tree finding algorithm to achieve the fractal compression [10]. Based on the spatial correlation on the range and domain blocks, Truong et al. proposed searching the matching domain block from the adjacent domain block of the current range block [11]. Chen et al. used normalized one-norm and kick-out condition to encode images, which had 22% execution time improvement ratio in average when compared with the traditional method [12]. Amol et al. extracted image feature for the encoding of fractal image, which reduced encoding-decoding time and achieved a good quality of compressed image [13]. Also, Schwartz et al. presented a scheme based on robust feature descriptors to speed up the encoding time [14]. In order to reduce the encoding time, Doudal et al. proposed a faster method by reducing the size of the domain pool, which is based on the lowest horizontal and vertical DCT coefficients of domain blocks, and combined their method to the AP2D approach which uses two domain pools in two steps of encoding [15]. All these methods have a common idea that the best matching domain block is sought in the original images. However, when the image size grows the number of the domain block for matching will increase by multiples, making the computation more time-consuming. In this paper, we propose a new fractal coding method, called fractal dictionary coding (FDC), based on the pre-defined dictionary. The paper is organized as follows: In Section 2, we introduce the theory of traditional fractal coding algorithm. In Section 3, we introduce our algorithm. Then the experimental results are demonstrated in Section 4, which is followed by conclusions.

## The Traditional Fractal Coding Algorithm

**Figure 1:**
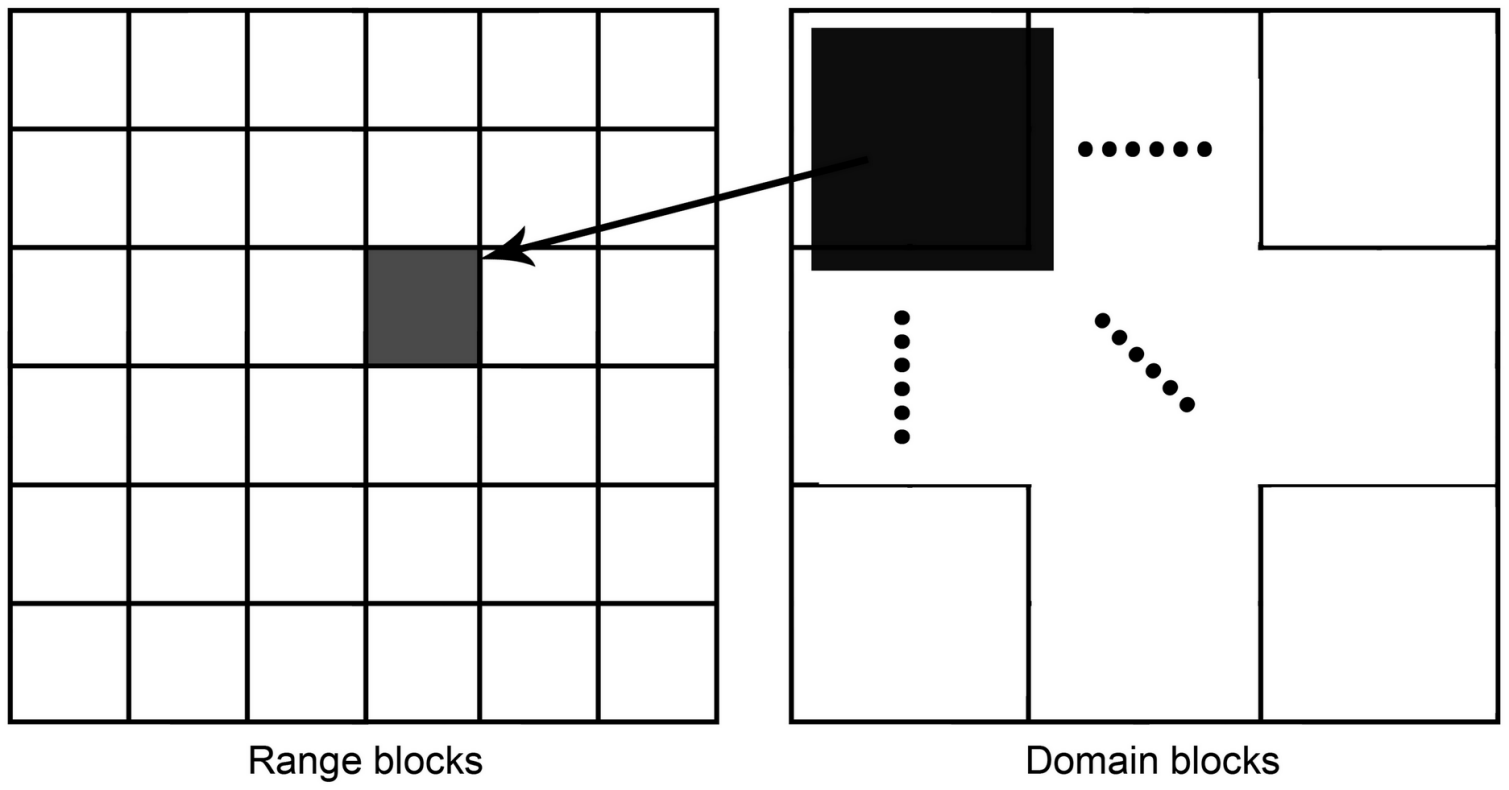
One mapping from a domain block to range block [16].

Fractal coding is based on Iterated Function System. An image is first partitioned into non-overlapping cells as range blocks. Then, the image is also divided into overlapping sub-blocks, which are named as domain blocks. Each range block is mapped from one of the domain blocks (See Figure 1 [16]).

Generally, the domain block has double size of the range block. For each domain block, we map a square domain cell to a square range cell [1] by Eq. (1), where D is the domain block, 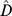 is the mapped domain block, and *k* and *l* are the horizontal and vertical cell position in image, respectively.
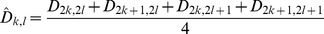
(1)


To find a best matching domain block, a range block has to be compared with each linear processed domain block to record the minimum distortion. The linear processing includes eight affine transformations. These transformations do not modify pixel values; they simply shuffle pixels within a range block, in a deterministic way. Besides, we denote *d* as the distortion criterion between the range block and the transformed domain block. See Eq. (2).
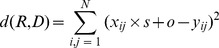
(2)where *N* is the pixel number of a block, *s* is contrast factor, *o* is luminance factor, *x_ij_* represents the value of pixel (*i*, *j*) in range block *R* and *y_ij_* represents the value of pixel (*i*, *j*) in affine transformed domain block *D*. After comparisons with the range block, a transform minimizing the distortion between the range block and the selected domain block is recorded.

In the coding process, the domain block with the minimum distortion *d* is regarded as the proper one to match the range block. Applying the Least-Square Method on Eq. (2), *s* and *o* can be calculated according to the minimum distortion *d*, respectively. The Equations are defined as follows:
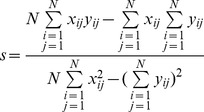
(3)

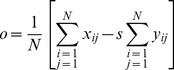
(4)


After encoding the image, the best domain block’s position (*i*, *j*), *s*, *o* and *t*, five parameters totally, are stored.

The decoding operation simply iterates on an initial image with encoding parameters, defined as Eq. (5), until converging to the final decoded image. The initial image *D*_0_ can be an arbitrary image with the same size as the encoded image.
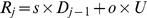
(5)where *D_j–_*_1_ represents the domain block at the (*j*–1)-th iteration, *U* is a matrix with the same size as *D_j–_*_1_, whose elements are all 1 and *R_j_* represents the result of the *j*-th iteration. In general, the process should be in 7 to 8 iterations to obtain a good decoded image.

## The Proposed Method

For the traditional fractal encoding, a matching procedure with domain blocks is very time consuming. It is a challenge to reduce the comparison number. In traditional method, each image has its own domain block pool, which means the domain block pools may be different if the images are not the same. Due to the fact that domain blocks cannot be reused, it increases the extra computation time. There is also another problem that the number of domain blocks increases quickly with the image size growing. What’s more, in the decoding process the iteration has to be repeated several times to reduce the decoding error. As Ozawa [17] shows that every two images can be used as the other’s domain block pool and can be mutually encoded, we believe that there is also a pair of relations between an image and a fractal image. With this mind, we establish a codebook, which is called dictionary in the algorithm. For a range block in image, we can search a matching domain block in a public domain block pool. Once the distortion criterion satisfies Eq. (6), the best matching domain block is found.
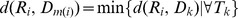
(6)where *R_i_* represents the *i*-th range block in the original image, *D_k_* is the *k*-th domain block in a BTC (Block truncation coding [18]) queue, which will be discussed in Section 3.1, and *D_m(i)_* is the domain block that minimizes the value of *d* with the range block.

In the decoding process, the reconstruction is completed by calculation with the domain blocks in the dictionary only once. Thereby, the decoding process operation can be accomplished quickly and has no iterative errors.

### 3.1 Block Truncation Coding

Delp et al. [18] presented a Block truncation coding (BTC) scheme for image compression. It is a type of lossy image compression technique for grayscale images. In this method, each block can be converted a BTC value. Firstly, the original image is divided into non-overlapped blocks. For each block, one pixel in it is represented by one bit. As shown in Eq. (7), if the pixel value is greater than or equal to the average value of the block *X_ave_*, then we set the pixel as 1, otherwise set the pixel as 0.
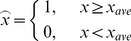
(7)


**Figure 2:**
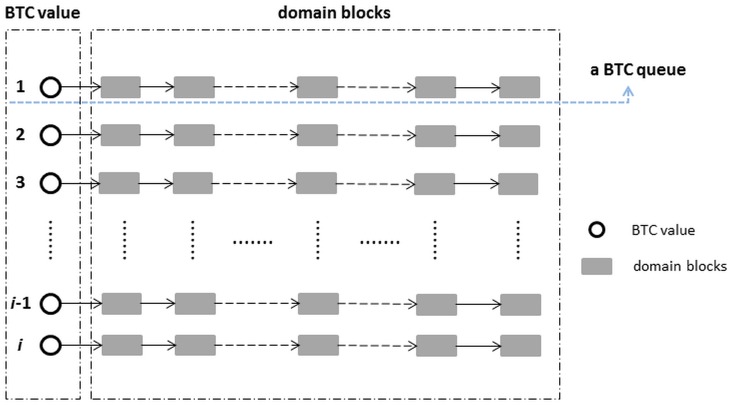
The structure of fractal dictionary.

We can treat this matrix as a vector with a binary sequence and calculate its decimal number which is called BTC value. Because each domain block owns a unique BTC value and a BTC value can be shared by a series of domain blocks, the BTC value can be treated as a classifier for the domain blocks. Before generating a dictionary in our algorithm, each domain block is classified by the BTC value, i.e. the domain blocks sharing the same BTC value are added to the corresponding BTC queue, as shown in Figure 2.

In the classifying process, the matching norm *d* is calculated between each block. The block will be added into the dictionary either if the norm value is greater than a threshold or if the number of the blocks with the same BTC value is less than the upper limited number set in the algorithm.

### 3.2 Fractal Dictionary

As mentioned above, ffractal dictionary (FD) is a set of domain blocks. Obviously, for various kinds of images, fractal dictionary with rich domain blocks contents can help achieve good coding results. These rich contents characterize comprehensive image patch features, such as edge features, smooth features and texture features. To obtain abundant image blocks, we utilize the Mandelbrot sets and Julia sets (abbreviated as M sets and J sets) to generate the dictionary. M sets and J sets are the classic fractal images. They have rich information (see the discussion in Section 4.4.3), and can display the embedding topological structures in different scales.

**Figure 3:**
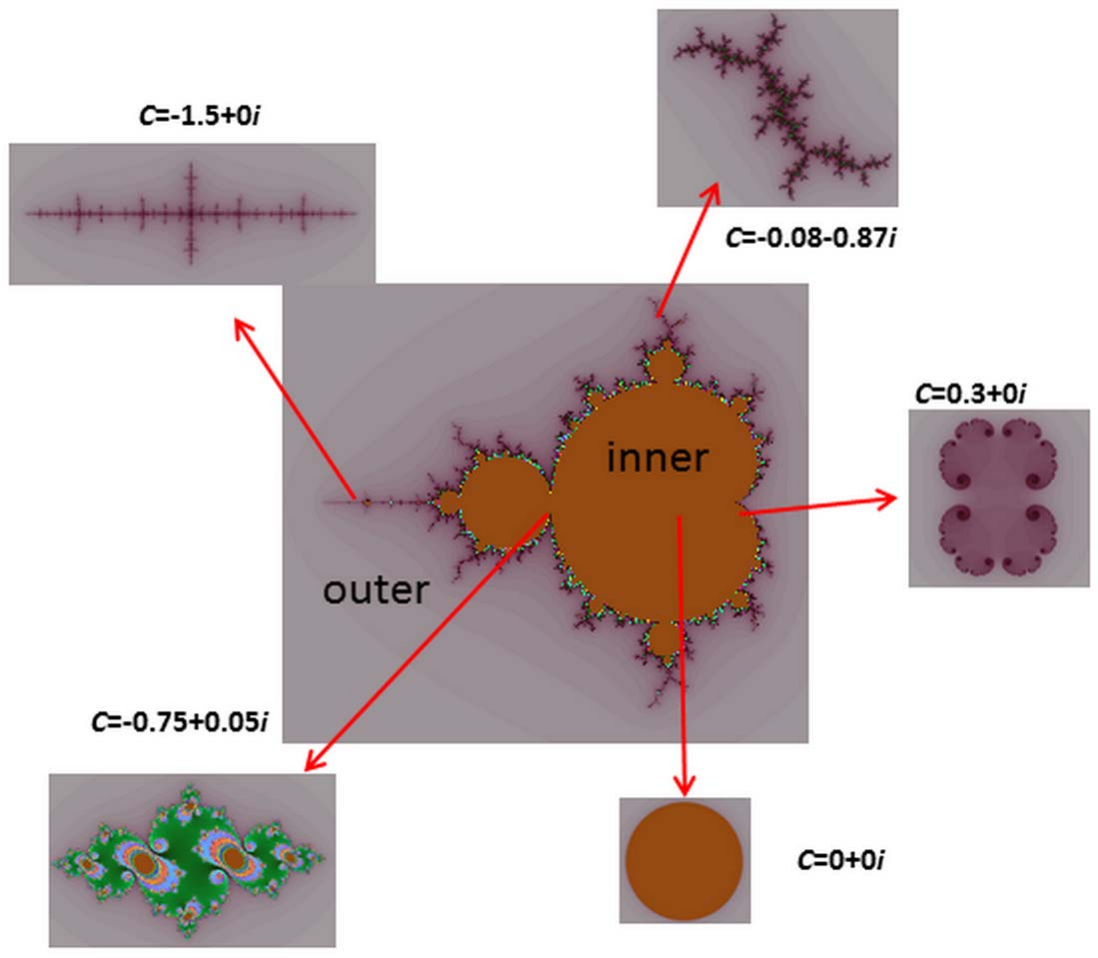
The relationship between Mandelbrot set and Julia sets.

Take the mapping function *f*(*z*) = *z* ^2^+*c* for instance, the M set records the value of a unique *c* under the iteration regulation *z_n_*_+1_ = z*_n_* ^2^+*c*. The J set is a result of a fixed *c* value with a location *z* under the same regulation, which is depicted in Figure 3. The points in the M set and at the border of it are converged, while the outside sets are diffused.

When selecting a converged point from M set, we can easily construct a J set. As Figure 3 shows, the J sets are various when *c* values are selected differently. In our research, all the converged points from the M set are selected to construct the J sets whose the image blocks are prepared for generating a dictionary. The details are as follows:

1. **Obtain parameters of J sets.** The first step is to create the standard M set image of the *W×W* size. We choose the converged points from M set image, denoted by *Φ_N_*, where *N* is the number of the selected points.
2. **Construct J sets.** For each point in *Φ_N_*, we can get the unique *c* value and create the corresponding J set of *W×W* size by the escape time algorithm [19]. In the algorithm, the escape time is recorded as pixel value, denoted by *V*_(*k, l*)_, where (*k, l*) represents the pixel coordinates (*k, l*). All the values should satisfy Eq. (8).
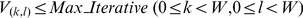
(8)where Max_Iterative represents the maximum number of iterations.
3. **Preprocess fractal image.** Since the values of escape time may not fall in [0, 255] and *V*_(*k, l*)_ is likely small, they are multiplied by *H* for normalization, as shown in Eq. (9). *H* can be an arbitrary integer, but it would be better to be a prime one larger than 256. The prime numbers are indivisible by any other numbers, so the process shown in Eq. (9) may lead to more even distribution of block values. In our experiments, *H* is 479. Repeat this step on different *H* to generate a variety of fractal images.
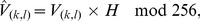
(9)Note that 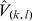 is the value in (k, l) after normalization and 256 is the color level of greyscale image.
4. **Generate domain blocks.** After the preprocessed J set are constructed, we regard the J set as a domain block pool and divide them into domain blocks, as depicted in Figure 1.
5. **Classify the domain blocks.** As Section 3.1 discussed, we construct the fractal dictionary. The details are shown in Figure 2.

Once fractal dictionary is generated, it should be optimized to make the dictionary rich enough before directly using for coding. The following aspects should be considered.

- **The number of domain blocks in a BTC queue.** First, a BTC queue should have enough blocks. Only in this case, it can be fully guaranteed that the image can find a suitable domain block in the dictionary. However, if the dictionary has too many blocks, the speed of the coding will slow down, which will be discussed in Section 4.5. In our experiment, the size of a BTC queue is 10.
- **The number of redundancy blocks in dictionary.** Due to the similarity of the M-set image, the dictionary also includes a large number of similar blocks, which are considered as duplicate blocks. Obviously, these duplicate blocks should be removed from the dictionary. A BTC queue in dictionary should consist of *K* domain blocks. Initially, the block can be added to the queue, if it has more than 30 distortion with other blocks, where the 30 is an estimated value. In the optimization process, each image block in the dictionary is multiplied by a large prime number (similar with Eq. (9)), producing a new domain block. In our experiments, the prime numbers are 4273, 4283 and 4289, respectively. Then BTC value of the new domain block is recalculated. If the new BTC value does not equal to its former value, we recalculate all of the distortions using Eq. (2) with the domain blocks in the new BTC queue, including the new domain block, and replace a domain block with the minimal distortion with the new one.

### 3.3 Image Coding

Once the fractal dictionary is constructed, the image can be encoded as follows:

1. **Load the fractal dictionary.** Each domain block is defined by *D_i_* and the set of all domain blocks is defined by *D_L_*, where *L* is the number of blocks.
2. **Partition the image.** The image is divided into non-overlapping range blocks, which is denoted by *R_i_*_._ *R* represents all range blocks. Obviously *R* covers the whole image.
3. **Search the best matching domain block.** Denote *D_m__(i)_* as the best block matching with *R_i_*, which has the minimum value of 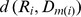. The process is as follows.
  1. Calculate the BTC value of range block and locate the corresponding BTC queue.
  2. Calculate the distortion metric. From the above analyses, the block with affine transformation (*t*) that minimizing the distortion metric by Eq. (2) is the best matching domain block. Record its position (*offset*) in the BTC queue, the contrast factor (*s*) and the luminance factor (*o*) and the affine transformation (*t*).

Finally, each range block of the encoded image is stored in the format of (*BTC*, *offset*, *t*, *s*, *o*). In the proposed algorithm, the searching process is just in a shared dictionary file. The dictionary file can be pre-loaded in the memory, reducing the reading time from the disk.

### 3.4 Decoding Process

Because the dictionary is fixed, the iteration in decoding process only executes once. The algorithm is described as follows:

1. **Load the dictionary.**
2. **Restore the original image.** For each dictionary code, we search the matching domain block in the dictionary by the *BTC* and *offset* parameters and use Eq. (5) to decode the image. Notice that *D_j–_*_1_ in Eq. (5) is the best matching domain block in the dictionary. The reconstruction only need one iteration, so *j = *1.

## Results and Discussion

### 4.1 Simulation Results

**Figure 4:**
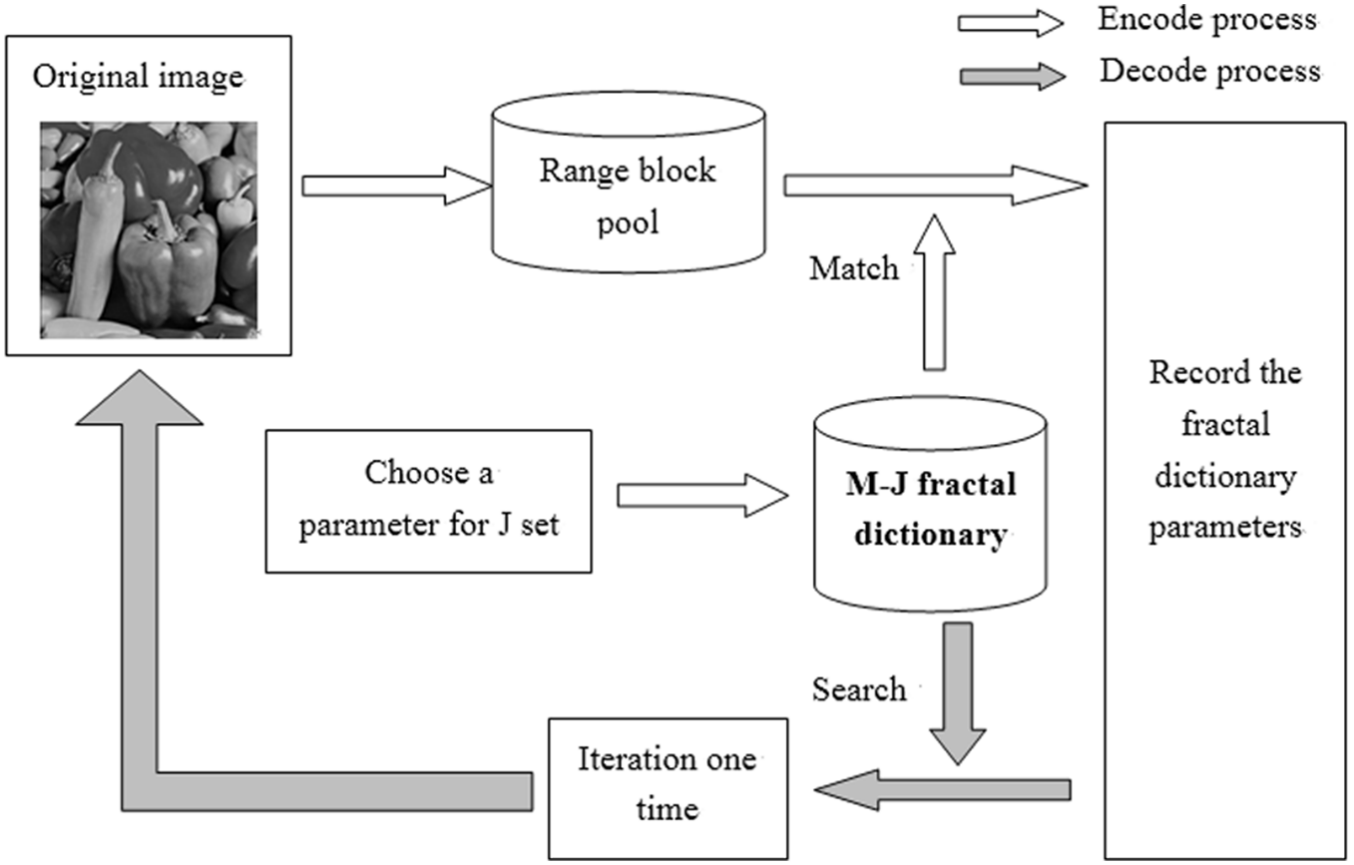
The coding-decoding system.

We use the Miscellaneous [20] as our database, which consists of 16 color images and 28 monochrome images. The compression system is shown in Figure 4. All experiments were conducted on a Core(TM) i5(2.40 GHz) PC. In the experiment, the size of the M set and the size of J set are both assigned as 256. The mapping function of both M set and J set are *f*(*z*) = *z* ^2^+*c*. We construct various kinds of J set based on the converged points in M set.

The J sets are divided into 8×8 block, all of which are used Eq. (1) to regulate a 4×4 block, so that encoding time can be reduced when compressing an image. The sixteen pixel values in a domain block with binary format can be converted to a decimal number–BTC value, ranging from 0 to 65535. Due to the fact that all pixel values are not smaller than the average value, so the minimum number 0 doses not exist. Although all pixel values are not able to be bigger than the average, they maybe equal to the average value. According to Eq. (7), the maximum number can be 65,535. The number of blocks *K* with the same BTC value is assigned as 10 at most, thus the final dictionary used in the experiments contains (65,536−1)×10 = 655,350 domain blocks. Moreover, for arbitrary blocks with the BTC value 65535, the matching norm value is 0, according to Eq. (2). It means that a block with BTC value 65,535 can represent all blocks with the same BTC value. Therefore the number of blocks in the directory is 655350−9 = 655341 in fact.

### 4.2 Compression Ratio

The traditional scheme needs five parameters for image reconstruction, that is domain block position (*D_x_*, *D_y_*), *s*, *o* and *t*. Suppose *s* and *o* are both assigned eight bits and affine transformation *t* is assigned three bits; for an image with 256×256 size, the domain block position is assigned eight bits to *D_x_* and *D_y_*, respectively. So the compression ratio is 3.66 when the range block size is 4×4. However, if the image size becomes larger, such as 512×512, the corresponding bit allocation for domain block position will be eighteen bits totally, and the ratio becomes lower, 3.46.

In the proposed method, a fixed domain block size, 4×4, is applied into the algorithm. The bit allocation for each parameter is shown in Table 1.

**Table 1:** The bit allocation for parameters.

Parameters	Bit allocation
BTC	16 bits
Block position	4 bits
*t*	3 bits
*s*	8 bits
*o*	8 bits

The corresponding compression ratio is (4×4×8)/(16+4+3+8+8)≈3.28. As we do not record the domain block position, the compression ratio does not change for different size. In addition, two bits can be allocated for quantization of s, which has four quantized value {0.25, 0.5, 0.75, 1} with approximately zero quantization error [21]. In this way, the ratio is up to (4×4×8)/(16+4+3+2+8)≈3.87. However, if we try to quantize the s and o too simple, the compression ratio would grow, resulting in a poor reconstruction quality. Besides, if we set a range block size 8×8, the corresponding BTC bit allocation would be 64, so the compression ratio is (8×8×8)/(64+4+3+8+8)≈5.89. From above analysis, in this fractal coding method, the domain block size determines the compression ratio when the sizes of other parameters are fixed.

### 4.3 PSNR and Time Consumption

PSNR (Peak signal-to-noise ratio) is commonly used to measure the quality of reconstruction for images. When comparing compression algorithms, PSNR is an approximation to human perception of reconstruction quality. It is defined as follows:
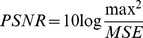
(10)where *MSE* is mean squared error between two images, and *max* is the maximum possible pixel value of the image. In this case, *max = 255*. Typical values for the PSNR in lossy image are between 30 and 50 dB, provided the bit depth is 8 bit, where higher is better. In addition, due to the encoding speed problem in fractal coding, time consuming is also another index that we should take into consideration. For a large-size image, the encoding process needs a long time. As discussed above, the performance can be evaluated by the time consumption and the value PSNR, two comparative indices.

**Figure 5:**
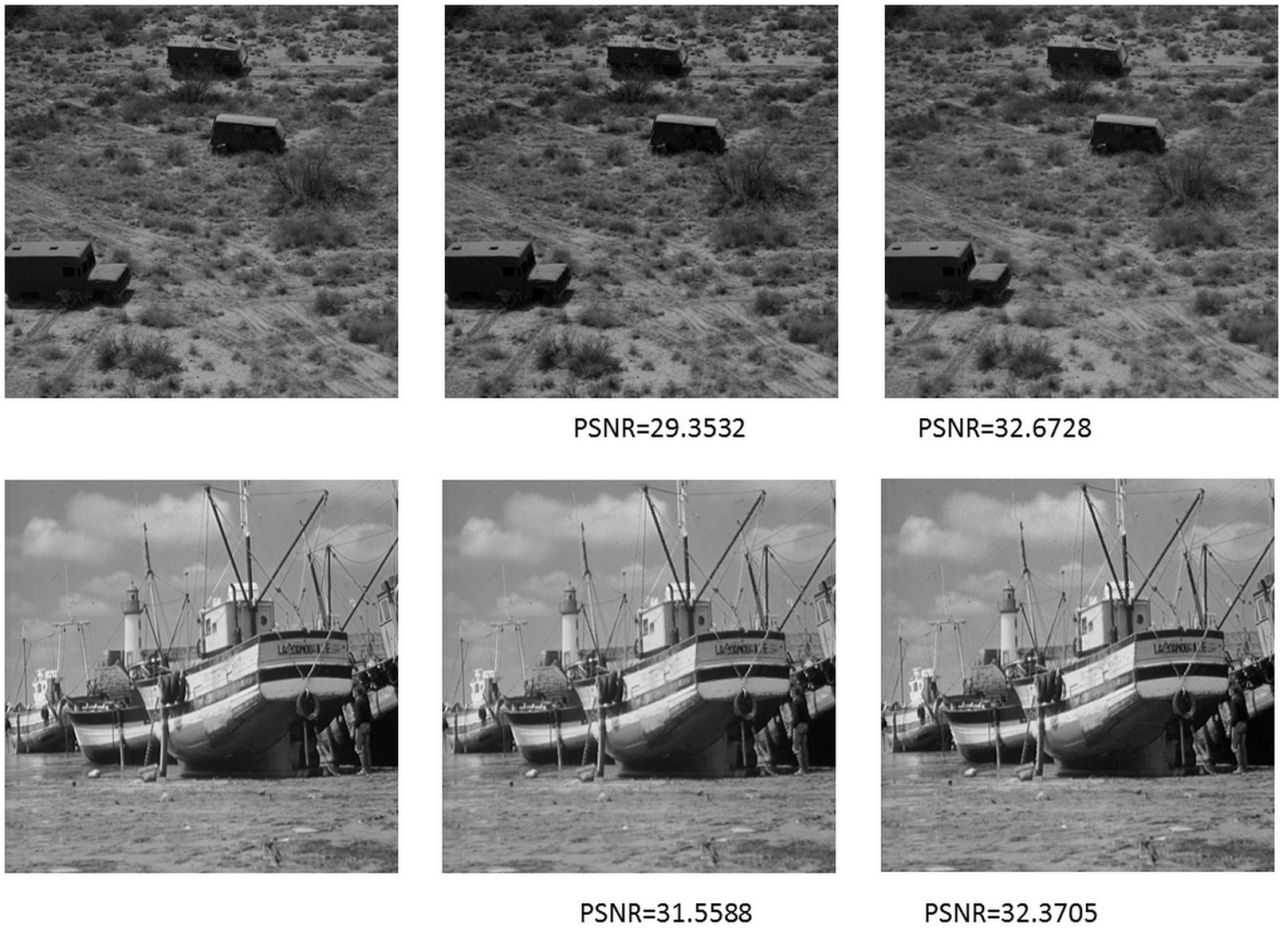
The original and decoded images (The images in first column are original images, the images in second column are the decoded images by TFC, and the images in last column are the decoded images by FDC).

**Figure 6:**
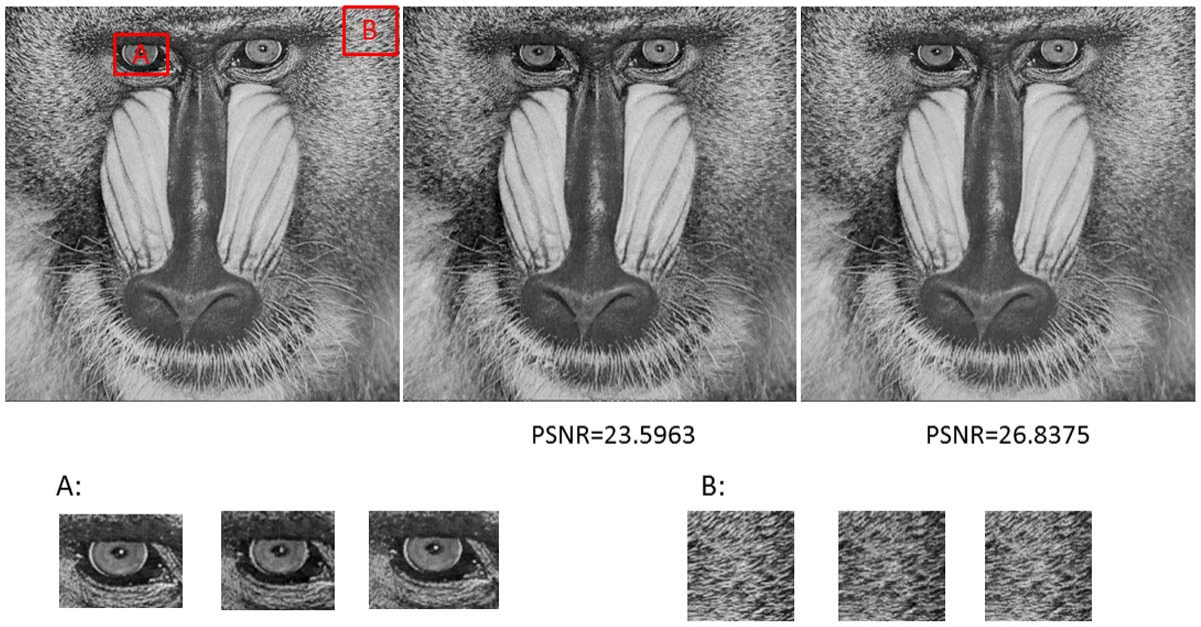
The decoded images of Baboon and its enlarged region comparison.

Figure 5 shows the decoded images by fractal coding algorithm (TFC) and our proposed algorithm. Figure 6 is the decoded images of Baboon, and the second row is the comparison regions of region A and region B. It can be seen from Figure 6 that the TFC is failed with dealing with the patches with details, because, as we can see from region A and region B, its recovery is blurring.

### 4.4 Comparison of Other Scheme

In this section, we compare our proposed method (FDC) with five schemes, which are traditional fractal coding (TFC), EP_NRS [22], mutual coding algorithm [17], random sequence dictionary based encoding and VQ method. The experimental results show that the FDC works better both in image reconstruction and in reduction of encoding time.

#### 4.4.1 Comparisons with TFC

We calculate PSNR and the time consumption of Lena image, Peppers image, Elain image and the Baboon image with the same size of 256×256. The results are shown in Table 2. It is obvious that FDC is 158 times quicker than TFC at least, saving plenty of time, and the value of PSNR is almost larger than 30, which is also higher than the former. The experimental results can validate the efficiency of the proposed algorithm. By using FDC, the coding efficiency improves greatly. The computational complexity of the traditional fractal encoding is O((*n*/4)×(*n*/4)×(*n*-*K*+1)×(*n*-*K*+1))∼O(*n* ^4^) for a *n×n* image when the size of domain block is *K*. While the computational complexity of our proposed method achieves 10×O((*n*/4)×(*n*/4))∼O(*n* ^2^), which has an obvious advantage over the traditional method. Therefore, it shows that the FDC can achieve a good coding performance and has a great advantage in coding time.

**Table 2:** The comparison of four different images between TFC and FDC.

	Lena		Peppers		Elain		Baboon	
	Time (seconds)	PSNR	Time (seconds)	PSNR	Time (seconds)	PSNR	Time (seconds)	PSNR
TFC	45	29.165	47	27.878	47	31.2672	47	26.4865
FDC	0.226	32.014	0.266	32.726	0.297	32.748	0.359	28.2868

In addition, we test the time consumption and PSNR value of Lena images with different sizes and the results are shown in Table 3. With the size of the image growing, both the PSNR and the time consuming increase. However, the time consumption and the size of the image present a linear relationship. Table 3 shows that our method has a better reconstruction quality and the coding speed improves substantially.

**Table 3:** The results of our algorithm with different sizes of image.

	Value	576	640	704	768	832	896	960	1,024
FDC	Time	1.657	1.781	2.14	2.407	2.984	3.469	4.063	4.61
	PSNR	33.6180	34.1698	34.78448	35.06283	35.6322	35.9392	36.4240	36.65260
TFC	Time	629	965	1,412	2,012	2,613	3,501	4,007	5,013
	PSNR	33.1872	33.6285	34.42822	34.5644	35.07899	35.28248	35.66146	35.74925

#### 4.4.2 Comparisons with EP_NRS and Mutual Coding algorithm

We choose Lena image with the size of 512×512 and calculate the domain block number, the comparing block number, the coding time, and the PSNR with the literature algorithms, such as the traditional fractal coding (TFC), the EP_NRS algorithm [22] and the mutual coding algorithm [17]. The results are shown in Table 4. Considering the image size is 512×512 and the small size range block will significantly reduce the encoding speed, we select range block as size of 8×8 when applying the above three algorithms. The green channel of Lena image is processed in the experiments since it is similar with the greyscale Lena image. The experimental results of EP_NRS algorithm are obtained from Lin [22]. The efficiency of the mutual coding algorithm depends on the choice of range image, which could enhance the PSNR, but it does not reduce the coding time. The EP_NRS algorithm has an advantage in time consumption through classifying range blocks of the image. However, it search domain blocks in a relatively comprehensive and strict way, causing the PSNR value decreasing correspondingly. In our proposed algorithm, the PSNR value is competitive. What’s more, the comparing block number is the smallest, so that it could reduce the coding time greatly. The speed of FDC algorithm is 400 times faster than that of TFC algorithm and 100 times faster than that of EP_NRS algorithm. In conclusion, the proposed algorithm based on the fractal dictionary can achieve a good performance in both reconstruction quality and time consumption.

**Table 4:** The four different code algorithms with the image of Lena.

	TFC	Mutual codingalgorithm	EP-NRS	FDC
Parameter		Elaine		
Number of domain block	247,009	247,009	247,009	655,341
Number of compare block	8,093,990,912	8,093,990,912	11,508,128	163,840
Coding time	401	400	59.24	1.047
PSNR value	32.5445	31.6168	31.59	33.3579

#### 4.4.3 Comparison with Random Sequence based Dictionary

Considering that the computer can neither produce a large amount of random sequences nor produce a large amount of random blocks with BTC classification at one time, we present the following algorithm to construct random sequence based dictionary:

Step 1: Generate an array of 256 numbers ranging from 0 to 255 randomly.
Step 2: Randomly produce a number as the array index to indicate the number in the array.
Step 3: Repeat step 1 and step 2 sixteen times to get a 4×4 size block. Calculate its BTC value and add the block to the corresponding BTC queue. If the BTC queue is full, then calculate the distortions between the new block and the other blocks in the BTC queue, replacing the domain block minimizing distortion with the new one.
Step 4: Repeat step 1 to step 3, until the number of the blocks in the dictionary reaches 10×65535 (block number in BTC queue multiplied by the number of BTC values).

**Figure 7:**
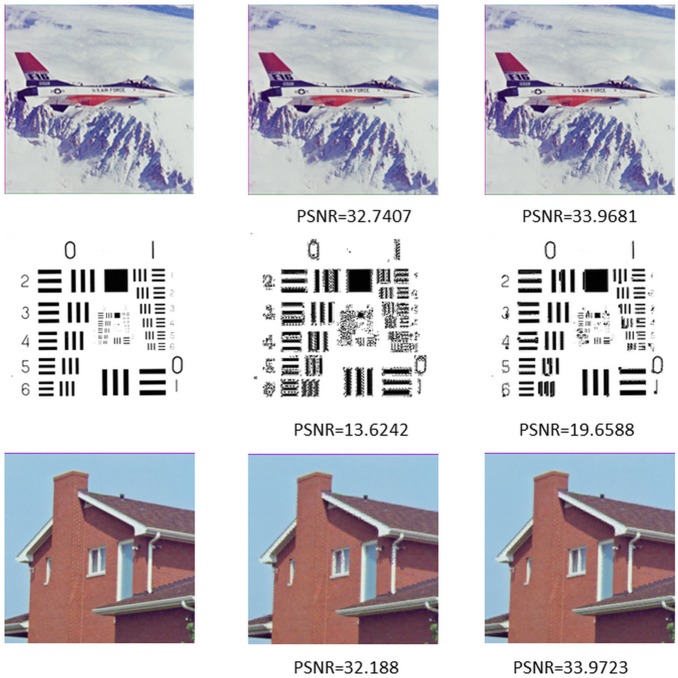
The images in first column are original images, the images in second column are the decoded images by random dictionary, and the images in last column are the decoded images by FDC.

Figure 7 is the comparison between the random dictionary and fractal dictionary. As Figure 7 shows, both random dictionary and fractal dictionary have a good recovery performance in smooth patches and texture patches. However, when it comes to apparent edge blocks, the fractal dictionary does better.

**Figure 8:**
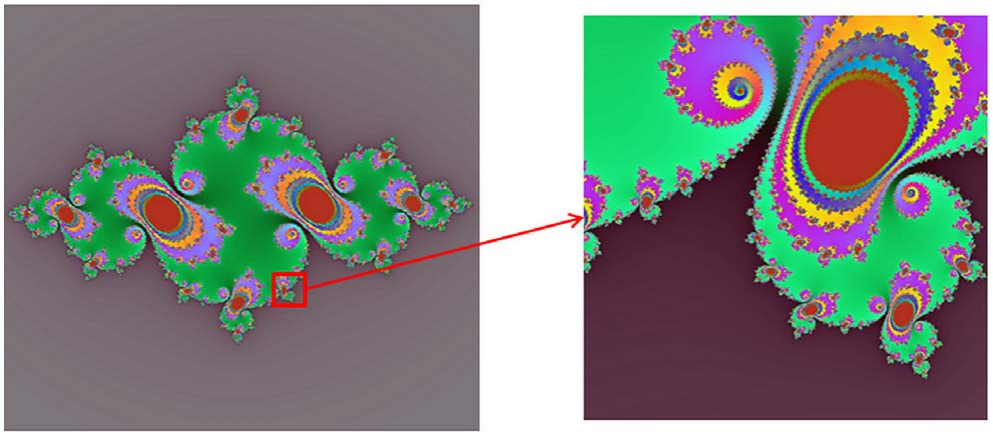
the Julia set and its bottom right part.

In order to demonstrate the proposed method has a good quality in apparent edge recovery, we select a J set with mapping parameters *c* = –0.75+0.05×*i* and enlarge its bottom right part (see Figure 8). From the left one, we can easily see that the J set contains apparent edge patches and smooth patches. From the right one, we can see the border of the J set is not smooth, which contributes to restoring the texture patches in the image. So we deem the J sets have rich information. Based on the above discussion, we arrive at the conclusion that a suitable domain block from J sets with a transformation that minimizing the distortion can be found to map with the range block in images. That is to say, it is able to encode an image by the J sets. However, for a random sequence, it is hard to present an apparent and smooth edge.

#### 4.4.4 Comparisons with VQ method

In addition, we use the Lena image and Baboon image as training sets, constructing a VQ (Vector Quantization) codebook with 256 vectors based on LBG [23]. The details are as follows: Codebook1 is trained on both Lena and Baboon image, Codebook2 is trained only on Baboon image and Codebook3 is trained only on the Lena image.

As the proposed method has extra affine transformation calculation processes, the distortion between the range block and domain block becomes more accurate, which contributes to the good reconstruction quality. Table 5 shows that our proposed method achieves better performance than the VQ scheme.

**Table 5:** The PSNR values on different codebooks.

	Codebook1	Codebook2	Codebook3
Lena	29.2207	27.667	30.4284
Baboon	22.9686	23.8538	22.40464

In the end, we make a comparison with VQ method, TFC method and FDC method, shown in Table 6. Also, Table 7 lists the PSNR values of the whole dataset with random sequence based dictionary method, VQ method, TFC method and FDC method.

**Table 6:** comparisons of the features of three algorithms.

VQ [1]	TFC	FDC
• Codebook	• Virtual codebook	• Codebook
1 Training set of images	1 Contracted and processed domain blocks extracted from original image itself.	1 655,341 pre-defined domain blocks in the dictionary.
2 Codebook design		
3 off-line transmission of codebook.		
• **Block matching**	• **Encoding of range blocks**	• **Block matching**
1 Selection of a block distortion measure.	1 Eight types of block matching in virtual codebook.	1 Use of the notion of block classification
2 Use of the notion of block classification.		2 Eight types of block matching in the dictionary.
• **Decoding**	• **Reconstruction, decoding**	• **Reconstruction, decoding**
1 Image code: list of addresses of blocks in the codebook.	1 Fractal code: list of block transformations consistent with an image partition.	1 Image code: list of addresses of blocks in the codebook
2 Direct reconstruction by look-up table.	2 Iterative reconstruction.	2 One iteration reconstruction.

**Table 7:** The PSNRs based on different schemes.

Image	The random dictionary	The VQ method	The traditional fractal coding method	The fractal dictionary coding method
4.1.01	32.9508	28.8084	31.2508	33.8970
4.1.02	33.6587	28.5788	31.0644	34.7343
4.1.03	28.6114	29.8671	33.0546	30.5313
4.1.04	29.8793	27.2766	32.4935	34.9144
4.1.05	32.188	27.7501	31.4542	33.9723
4.1.06	28.0582	24.7818	28.4162	29.4287
4.1.07	35.1906	32.2591	35.1626	36.6684
4.1.08	32.6441	30.2591	34.7663	34.2784
4.2.01	35.8855	27.8957	32.0995	37.8125
4.2.02	24.1887	21.3258	29.4128	28.9827
4.2.03	26.5486	22.9686	24.1815	27.2716
4.2.04	33.1018	29.2207	32.9591	34.3344
4.2.05	32.7407	28.5108	32.3286	33.9681
4.2.06	29.8416	26.1272	29.2448	30.7932
4.2.07	33.0054	28.3255	30.9864	33.9670
5.1.09	33.3843	29.3044	31.0650	33.9871
5.1.10	26.9131	23.3647	27.1571	28.0378
5.1.11	34.7241	28.5823	29.4359	36.4486
5.1.12	30.9467	26.0122	31.8889	31.3797
5.1.13	13.6242	15.2228	26.8759	19.6588
5.1.14	27.2907	25.741	29.4699	29.7049
5.2.08	30.4794	26.0937	27.31845	32.5446
5.2.09	27.1604	24.2871	29.8300	28.5566
5.2.10	27.3809	24.3548	28.22573	29.1839
5.3.01	32.2192	28.2891	31.7512	33.2846
5.3.02	30.5864	27.0437	29.2776	31.4943
7.1.01	34.6682	30.1697	32.8052	35.5746
7.1.02	37.8438	33.0943	34.2880	39.1784
7.1.03	34.9902	30.1818	30.8735	35.7599
7.1.04	35.6037	30.649	32.4829	36.5579
7.1.05	31.8108	27.6967	29.3764	32.5732
7.1.06	31.8998	27.785	29.3532	32.6728
7.1.07	32.9500	28.598	28.6803	33.6505
7.1.08	36.7386	31.7021	31.8649	37.5214
7.1.09	33.1243	28.6222	29.2926	33.8955
7.1.10	35.54232	30.8619	32.2126	36.4187
7.2.01	37.8172	32.6657	28.3818	38.2911
Boat.512	31.2312	27.4609	31.5588	32.3705
Elaine.512	34.0784	29.7331	29.0331	34.9626
Gray21.512	33.5643	27.531	34.8327	63.6233
house	30.3951	26.7953	30.3631	31.6885
Numbers.512	22.1529	18.8768	20.9069	25.8782
Rule.512	13.9936	11.852	13.8835	28.4160
testpat.1 k	21.9491	18.2474	18.8405	26.54797

### 4.5 The Number of Blocks in a BTC Queue

A BTC queue should have enough blocks for matching the range blocks, but too many blocks will reduce the speed of the algorithm. Suppose that if a BTC queue has very few blocks, the range block would be hard to search a suitable domain block as well as the transformation that minimizes the distortion. On the other hand, if a BTC queue has too many blocks, the searching will become time consuming, because it has to calculate the distortion between each domain block in the queue with the range block to get the closest one. In the experiment, we test the 256×256 Stream and bridge image. This image has many texture patches and texture patches are various. Therefore, it should have enough domain blocks preserved in the dictionary so that the range block with texture patches can find a suitable domain block with the transformation minimizing the distortion, while the smooth patches do not need many domain blocks for matching. The number of domain blocks in a BTC queue impacts on the restoration quality of the image with a certain amount of texture patches. In our experiments, we also test the 256×256 Lena image and the 256×256 Clock image, both of which have many smooth patches.

**Figure 9:**
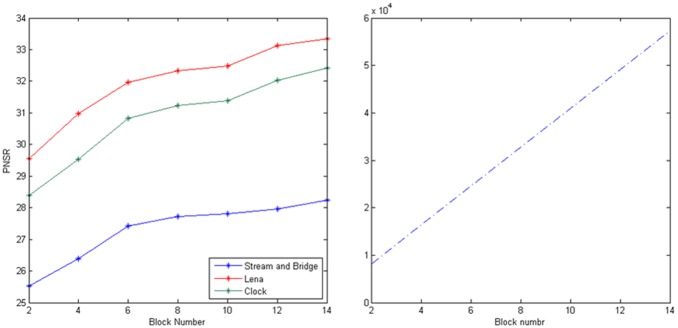
The PSNR and the compared block number when the number of block in a BTC queue are 2, 4, 6, 8, 10, 12, 14.

From Figure 9, we can see that when the block number increases, its PSNR grows. However, its comparison time is in linear growth, which means the time in encoding increases at the same time. In the experiments, we select 10 domain blocks in a queue as a compromise proposal.

## Conclusions

This paper has presented a new method of fractal image coding. It was based on a fractal dictionary, consisting of rich domain blocks generating from J sets. An image range block can be matched with the best- matching block in the dictionary by less comparison without losing the reconstruction quality. Experimental results show that the block number for comparisons during coding was obviously less than literature algorithms, which could explain why our algorithm is faster than other schemes. In addition, the PSNR is satisfying. Therefore it could give a good reconstruction quality when a fixed fractal dictionary is adopted. What’s more, the performance of the proposed algorithm has a superiority of the speed, especially for the large size images.

For future work, we will consider the bit allocation for coding parameters to improve the compression ratio and try to make the fractal dictionary adaptive to different size of domain blocks.

## References

[1] Jacquin AE (1990) A novel fractal block-coding technique for digital images. Acoustics, Speech, and Signal Processing, 1990. ICASSP-90. 1990 International Conference on. IEEE. 2225–2228.

[2] Barnsley MF, Jacquin AE (1988) Application of recurrent iterated function systems to images. Visual Communications and Image Processing’88: Third in a Series.: 122–131.

[3] Jacquin AE (1992) Image coding based on a fractal theory of iterated contractive image transformations. Image Processing, IEEE Transactions on 1: 18–30. 10.1109/83.128028 18296137

[4] Barnsley MF, Hurd LP (1992) Fractal image compression, AK Peters.

[5] Pi M, Mandal MK, Basu A (2005) Image retrieval based on histogram of fractal parameters. Multimedia, IEEE Transactions on 7: 597–605.

[6] Wang J, Zheng N (2013) A Novel Fractal Image Compression Scheme with Block Classification and Sorting Based on Pearson’s Correlation Coefficient. Image Processing, IEEE Transactions on 22: 3690–3702. 10.1109/TIP.2013.2268977 23797251

[7] Li ZH, Qu XL, Dai MA (2010) Research and Implementation of Fast Image Fractal Coding Algorithm. Applied Mechanics and Materials 34: 1360–1364.

[8] Duh DJ, Jeng JH, Chen SY (2005) DCT based simple classification scheme for fractal image compression. Image and vision computing 23: 1115–1121.

[9] Jaferzadeh K, Kiani K, Mozaffari S (2012) Acceleration of fractal image compression using fuzzy clustering and discrete-cosine-transform-based metric. Image Processing, IET 6: 1024–1030.

[10] Sze CJ, Liao HYM, Fan KC, Chern MY, Tsao CK (1996) Fractal image coding system based on an adaptive side-coupling quadtree structure. Image and Vision Computing 14: 401–415.

[11] Truong TK, Kung CM, Jeng JH, Hsieh ML (2004) Fast fractal image compression using spatial correlation. Chaos, Solitons & Fractals 22: 1071–1076.

[12] Chen HN, Chung KL, Hung JE (2010) Novel fractal image encoding algorithm using normalized one-norm and kick-out condition. Image and Vision Computing 28: 518–525.

[13] Baviskar AG, Pawale SS (2012) Efficient Domain Search for Fractal Image Compression Using Feature Extraction Technique. Advances in Computer Science, Engineering & Applications. 166: 353–365.

[14] Schwartz WR, Pedrini H (2011) Improved fractal image compression based on robust feature descriptors. International Journal of Image and Graphics. 11: 571–587.

[15] Doudal S (2011) A reduced domain pool based on DCT for a fast fractal image encoding. Electronic Letters on Computer Vision and Image Analysis. 10: 11–23.

[16] Wohlberg B, De Jager G (1999) A review of the fractal image coding literature. Image Processing, IEEE Transactions on 8: 1716–1729. 10.1109/83.806618 18267449

[17] Ozawa K (2008) Dual fractals. Image and Vision Computing 26: 622–631.

[18] Delp EJ, Mitchell OR (1979) Image compression using block truncation coding. IEEE Trans. Commun. 27: 1335–1342.

[19] Mandelbrot BB (1983) The Fractal Geometry of Nature, New York: W. H. Freeman and Company.

[20] USC website. Available: http://sipi.usc.edu/database/database.php?volume=misc. Accessed 2014 Jun 12.

[21] Tong CS, Pi M (2001) Fast fractal image encoding based on adaptive search. Image Processing, IEEE Transactions on 10: 1269–1277. 10.1109/83.941851 18255542

[22] Lin YL, Wu MS (2011) An edge property-based neighborhood region search strategy for fractal image compression. Computers & Mathematics with Applications 62: 310–318.

[23] Linde Y Buzo A, Gray RM (1980) An Algorithm for Vector Quantizer Design, IEEE Trans. on Commun (28): 702–710.

